# Understanding the Awareness, Knowledge, and Opinion of Dairy Cattle Welfare among Dairy Farmers in Keningau, Sabah

**DOI:** 10.3390/ani11061750

**Published:** 2021-06-11

**Authors:** Mohammed Babatunde Sadiq, Sim Song-Lin, Siti Zubaidah Ramanoon, Sharifah Salmah Syed-Hussain, Wan Mastura Shaik Mossadeq, Mohd Shahrom Salisi, Rozaihan Mansor

**Affiliations:** 1 Department of Farm and Exotic Animal Medicine and Surgery, Faculty of Veterinary Medicine, Universiti Putra Malaysia, Serdang 43400 UPM, Malaysia; sadiquemohammed99@yahoo.com (M.B.S.); sramanoon@upm.edu.my (S.Z.R.); 2 Faculty of Veterinary Medicine, Universiti Putra Malaysia, Serdang 43400 UPM, Malaysia; ssl1996@gmail.com; 3 Centre of Excellence (Ruminant), Faculty of Veterinary Medicine, Universiti Putra Malaysia, Serdang 43400 UPM, Malaysia; 4 Department of Veterinary Clinical Studies, Faculty of Veterinary Medicine, Universiti Putra Malaysia, Serdang 43400 UPM, Malaysia; ssalmah@upm.edu.my; 5 Department of Veterinary Preclinical Sciences, Faculty of Veterinary Medicine, Universiti Putra Malaysia, Serdang Selangor, 43400 UPM, Malaysia; wmastura@upm.edu.my (W.M.S.M.); shahrom@upm.edu.my (M.S.S.)

**Keywords:** dairy cattle welfare, dairy farmers, knowledge, perception, veterinarian

## Abstract

**Simple Summary:**

Animal welfare is an important aspect that affects the health and productivity of dairy animals. This study reports the knowledge and opinion of dairy cattle farmers regarding dairy cattle welfare (DCW) in Keningau, Sabah. A total of 30 dairy farmers participated in the survey and the information collected includes their socio-demographic characteristics, knowledge, and opinions regarding DCW. Seventy per cent of the farmers (*n* = 21) had satisfactory-to-good knowledge of the DCW criteria, but their opinions differed regarding indicators of poor animal welfare. The understanding of DCW differed among farmers depending on the production level, educational status, herd size, and cattle breeds kept on the farm.

**Abstract:**

This study aimed to assess the knowledge and opinions about DCW among dairy cattle farmers in Keningau, Sabah. A questionnaire was developed, validated, and administered by hand to 30 farmers. The data collected include farmers’ and farm demographics, and opinions regarding the criteria and indicators of DCW. Only 17 respondents (57%) had heard of “dairy cattle welfare” before this study. Nine farmers (30.0%) had poor knowledge about DCW criteria, whereas 13 (43.7%) and 8 (26.7%) farmers had satisfactory and good knowledge, respectively. Farmers with higher education, larger herd size, high production level, and exotic cattle breeds showed a better understanding. Farmers understood most of the indicators; however, opinions regarding cattle behavior during milking, their physical appearance and their lying down behaviour need to be improved. Nevertheless, 28 respondents ranked their animals’ welfare as either good or satisfactory, which further reflects a poor implementation of DCW measures. The main factors suggested by farmers to influence DCW in their herds were facilities, worker issues, management practices, and animal well-being. In conclusion, guidance from veterinarians and animal welfare specialists may be needed to improve the farmers’ understanding and practices of DCW.

## 1. Introduction

One of the major public concerns regarding sustainable livestock farming is animal welfare [1], and societies have been pressuring farmers and shareholders in the livestock industry towards improved animal welfare [2]. The dairy industry is no exception in this regard, as the welfare of dairy animals remains an important aspect for preserving health and attaining better productivity [3,4].

Robust assessment methods are necessary to ensure animal welfare and to disseminate such information to farmers and consumers [5]. Several welfare assessment methods are available in the livestock industry, which are tailored toward specific management and farming systems. For instance, the European Food Safety Authority (EFSA) employed scientific opinion on the feasibility of existing welfare assessment methods in small-scale dairy farms (<75 lactating cows, family-run farms) and came up with a modified procedure of the Welfare Quality Protocol (WQ) [4]. Recently, the assessment of dairy cattle welfare (DCW) has been channelled towards the application of animal-based measures (ABMs), as welfare outcomes may vary in different management systems [5]. ABMs provide a direct indicator of animal welfare and how the animal copes under a specific farming system. Examples of ABMs include a body condition score, hock injuries, lying down behaviour, tick infestation load, California Mastitis Test score, physical injuries, locomotion scores and the animal flight zone [6,7].

Another vital aspect influencing animal welfare is the farmers’ knowledge and opinion about the subject. Several factors have been identified as drivers for dairy producers to improve their animals’ welfare [3,8]. This includes the desire to meet not only the demands of consumers and food retailers, but also public expectations of the proper treatment of dairy cattle [9]. In North America and Europe, most dairy farmers appreciate the need to ensure good comfort and welfare for their animals, and it was perceived as a priority in the industry [3]. Furthermore, management practices that are thought to influence animal welfare directly include calving management, nutritional management and housing environments [10,11]. For studies conducted among consumers, appropriate feeding, good stockmanship, and environmental cleanliness were stated as indicators of good dairy animal welfare [12,13]. Clark et al. [13] also found that the public was concerned about farm animal welfare in modern production systems, especially on naturalness and humane handling, in particular regarding the use of antibiotics as a prophylactic treatment.

In Malaysia, farm animal welfare is yet to attain the level of attention seen in other developed countries. Although the new Animal Welfare Act [14] was officially implemented in 2017, it was aimed at fostering more responsible pet ownership among Malaysians. Malaysia dairy farms are mostly characterized as indoor housing systems, with only a few farms providing pasture access for short intervals. However, confined housing and intensive dairy systems come with a lot of challenges that may predispose cows to poor welfare. This includes, but is not limited to prolong standing times, abrasive flooring, inappropriate stall designs, weather-related factors, poor hygiene, and lack of grazing area. Although there are published studies relating to DCW such as lameness prevalence and assessment of ABM on dairies [15,16], it is not known whether Malaysian dairy farmers are aware of, or have an understanding of, how modern farming practices may affect the productivity and welfare of farm animals. Thus, this study was conducted as a preliminary report assessing the knowledge, awareness and opinion regarding DCW among dairy farmers in Keningau, Sabah, Malaysia.

## 2. Materials and Methods

### 2.1. Study Design and Enrollment of Study Population

The study entailed a cross-sectional design and was carried out in Keningau, 95.2 km from Kota Kinabalu, Sabah, Malaysia. Farms were recruited from the list and contacts of dairy farms registered with the Department of Veterinary Services (DVS), Sabah, Malaysia. The inclusion criteria comprised location of the farms within the study state, a 5 km radius of the milk collection centre (MCC) of Stesen Pembiakan Ternakan (SPT) Sebrang, Keningau, which is presently producing dairy cattle, records on animal health and production, and where dairy cattle farmers were willing to participate in the survey. All farmers located within the specified region around the MCC were considered to be the target population. The location was selected for easy assessment and questionnaire administration since it served as the milk delivery point for most farmers in Keningau, Sabah.

### 2.2. Development of Instrument and Contents

A structured questionnaire was used in this study. The instrument was developed after reviewing related literature and published papers regarding DCW. Specifically, the Terrestrial Animal Health Code developed by the World Organization of Animal Health (OiE) and the Animal Welfare Enactment [14] were used as the main template in developing the instrument.

The questionnaire was structured into five sections. Section 1 was dedicated to obtaining respondents’ demographic information including gender, years of working experience, educational qualification, nationality, and age. Section 2 focused on the farm characteristics which included herd size, cattle breed, management system, availability of veterinary services, and average milk yield and production level. Management systems were categorized based on the provision of external grazing and pasture access (semi-intensive) or completely confined (intensive). The milk yield (production level) was used as the indicator of farm performance as described by Boniface et al. [17]. Farms producing an average of 10 L/cow/day were categorized as low producers, while those producing between 11 to 15 L and > 15 L/cow/day were considered medium and high producers, respectively.

Section 3 consisted of 18 items designed to assess farmers’ awareness and their opinion about the indicators of DCW. Farmers were asked if they had been exposed to the term “dairy cattle welfare”, whereas the other items were based on specific ABMs and indicators of DCW such as the presence of physical injuries, alterations in feed intake and body condition, isolation from herd mates, and lying down behaviour. Responses to items in Section 3 were presented using a dichotomous approach (yes or no).

Farmers’ opinions about DCW criteria were evaluated in Section 4. The items (*n* = 8) were selected from the common criteria employed on Canadian and American farms to assess the welfare of dairy cattle [11]. The items included mortality and morbidity rate, body weight, body condition, milk yield, cows’ response to human handling procedures, and complications from common procedures such as dehorning and treatment. A 5-point Likert scale ranging from strongly agree (score 5) to strongly disagree (score 1) was used to present the responses. In Section 5, farmers were asked to rank the welfare of their animals on a 10-point scale from 1 (very poor) to 10 (perfect) and to state the factors influencing animal welfare on their herds. The research team checked the items in each section of the questionnaire for validity and consistency. For ease of comprehension and to minimize challenges during administration, the questionnaires were first made available in English and later translated into Malay.

### 2.3. Administration of Questionnaire

The questionnaires were administered by a single researcher (S.S.L.) to each farmer in a paper format. The administration was conducted over 5 days (4–8 August 2019). Each respondent was allowed to select the preferred language (English or Malay), and those that experienced challenges either when attempting to answer the questions or had difficulty comprehending them were allowed to seek an unbiased explanation. Each participant responded to the questionnaire independently without any communication with the others.

### 2.4. Statistical Analysis

Data collected from the questionnaires were transferred into Microsoft Excel sheets for accurate and efficient data tabulation. IBM SPSS Statistics^®^ Version 25 was used to carry out the statistical analysis. Descriptive statistics were used to summarize the farmers’ and herd characteristics, and all the variables were categorized and presented as frequency distribution and percentages. However, the average milk yield was a continuous variable and it was categorized into 3 groups as described by Boniface et al. [17] with little modification. Descriptive statistics were also applied to present the number of responses to each item and the frequency distribution for dichotomous questions. Responses to Section 3 (indicators of DCW) were scored according to the provision of either correct (score 1) or incorrect (score 0) answers, and summated for each respondent. Thereafter, the cumulative knowledge score was computed for each respondent and checked for normality using the Kolmogorov–Smirnov test. The mean score was used to categorize the respondents into those having poor, satisfactory, or good knowledge about the indicators of DCW. A similar approach was used to compute and categorize the responses in Section 5. The association between farmers’ demographics and DCW knowledge was investigated using independent t-tests and one-way analysis of variance (ANOVA) depending on the number of levels or categories in each factor. A *p* value < 0.05 was considered for significant associations between the knowledge score and independent variables. Responses to items in Section 4 (DCW criteria) were presented using stacked bar charts. 

## 3. Results

### 3.1. Descriptive Results

The characteristics of the respondents and farm features are provided in Table 1. A higher proportion of the farmers were male (73%, 22/30), but only 6 (20%) were below 50 years old. As expected, most were Malaysians (83.3%, 25/30) and 77% (23/30) had secondary education. For the farms, most were managed intensively (90%), while similar proportions were classified as having a small (37%; 11/30) or medium (43%; 11/30) herd size. Fifty-three per cent (*n* = 16) of the farms were considered to have low-producing herds.

### 3.2. Farmers’ Awareness about the Term “Dairy Cattle Welfare” and Opinions Regarding DCW Criteria

**Figure 1:**
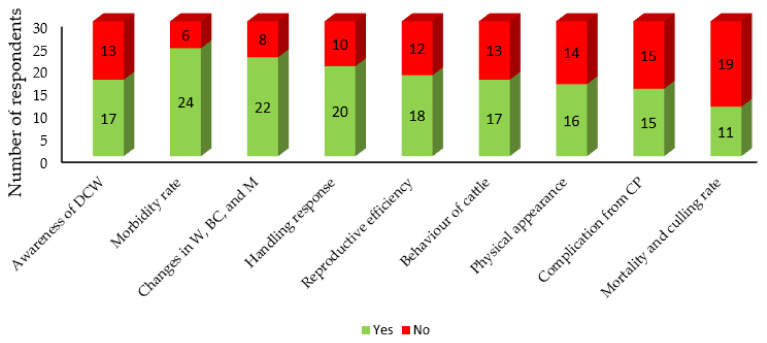
Number of respondents aware about the term “dairy cattle welfare” and responses regarding the criteria of dairy cattle welfare (DCW). **Note also:** weight, body condition, and milk yield (W, BC, and M); and common procedures (CP).

Respondents were asked whether or not they had heard of the term “dairy cattle welfare”. Only 17 (56.7%) had heard of the term before this study (Figure 1). Regarding the DCW criteria, the majority of the farmers selected morbidity rate (80%, 24/30), followed by changes in body weight, body condition and milk yield (73.3%, 22/30), and responses of animals to human handling (66.7%, 20/30). However, mortality and culling rate (36.7%, 11/30) and complications from common procedures (50%, 15/30) were the least considered criteria.

**Figure 2:**
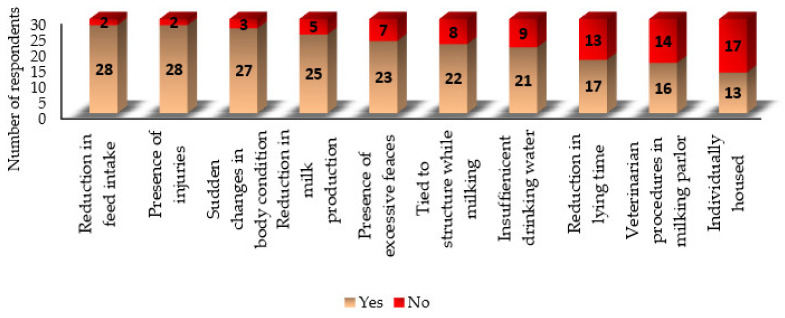
Responses on indicators of dairy cattle welfare.

Figure 2 shows the farmers’ responses to the other 10 items considered as DCW criteria. Poor indicators of DCW that were the most recognized by the farmers were the presence of injuries (93.3%, 28/30) and reduction in feed intake (93.3%, 28/30) followed by sudden change in body condition (90%, 27/30). The least recognized indicators were isolation of cattle (43.3%, 13/30), carrying out veterinary procedures in the milking parlour (53.3%, 16/30), and reduction in lying down time (56.7%, 17/30).

### 3.3. Farmers’ Knowledge about DCW Criteria and Associated Factors

The farmers obtained a mean (±SD) score of 12.1 (±3.47) out of 18 possible marks (Table 2). Nine farmers (30.0%) were considered to have poor knowledge about the DCW, whereas 13 (43.7%) and 8 (26.7%) farmers had satisfactory and good knowledge, respectively.

The mean knowledge score of respondents regarding DCW criteria was closely associated with level of education, breeds of cows kept, herd size, and production level (Table 3). Farmers with tertiary education had a significantly higher knowledge score (*p* = 0.029) compared to those with secondary education. Farmers with a large herd size (>100 cows) had significantly higher knowledge score relative to those with medium and small farms. Likewise, the knowledge score about DCW criteria was significantly higher among those considered as high producers compared to medium and low producing farmers. Farmers that had crossbreeds (Jersey x Holstein–Friesian and Holstein–Friesian x Sahiwal) had a significantly higher knowledge score (*p* = 0.03) than those who had local breeds (Sabah Friesian Sahiwal).

### 3.4. Farmers’ Opinion on the Indicators of DCW

**Figure 3:**
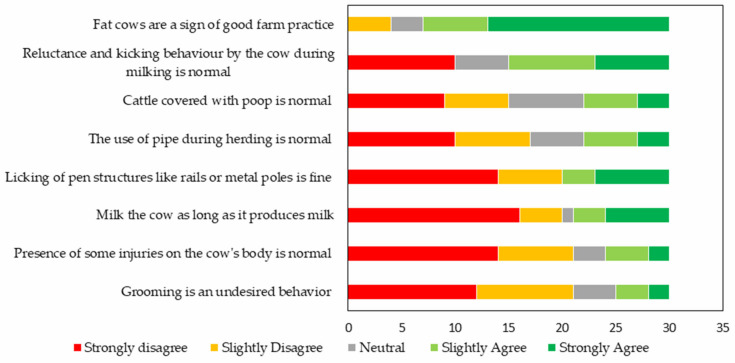
Respondents’ opinion on statements regarding inappropriate management practices and cattle behavior.

The majority of the respondents (60%, 18/30) disagreed with the statements about inappropriate practices and cattle behavior: 30% (9/30) gave a neutral response, and only 10% (3/30) agreed with the statements. Specifically, most of the respondents agreed with the statement, “Fat cows are a sign of good farm practice” and “Reluctance and kicking behaviour by the cow during milking is normal”. Meanwhile, the farmers slightly disagreed with the rest of the statements, but strongly disagreed with the statement, “Milk the cow as long as it produces milk”. Figure 3 shows the responses of the farmers arranged according to the proportions that disagreed with each statement. 

### 3.5. Farmers’ Ranking of on-Farm Animal Welfare Status and Factors Influencing DCW

Farmers were asked to rate the welfare of their animals on a 10-point scale and to state the factors responsible for the score selected. The mean (± SD) score was 8.0 (±1.83) (Table 4). A higher proportion (56.7%, 17/30) considered their animal welfare to be good, whereas 36.7% (11/30) and 6.7% (2/30) regarded theirs as satisfactory or poor, respectively. The majority of the farmers (33.3%, 10/30) mentioned facilities on the farm as the most important factor influencing animal welfare. Five farmers (16.7%) mentioned workers’ handling of animals, and three farmers (10%) stated management systems, whereas other factors presented by six farmers (20%) were categorized as miscellaneous.

## 4. Discussion

The proportion of respondents who either knew of or were ignorant of “Dairy Cattle Welfare” before this study was 56.7% and 43.3%, respectively. Most of the farmers had only secondary education, and this might explain their low exposure to DCW since the term is more likely to be introduced in tertiary schools. Among the categories employed in this survey to understand farmers’ current knowledge of criteria of DCW were morbidity rate, changes in body weight, body condition, and milk production level. Most of the farmers (80%) understood “morbidity rate”, whereas 50–60% of them selected either one or more of the latter indicators. Mortality and culling rate were least understood by the farmers as only 37% related the factors to DCW. By referring to the welfare code, mortality and culling can directly or indirectly indicate welfare status as it affects the productive lifespan of an animal. Mortality and a lower productive lifespan often result from the failure of animals to cope in an environment [18]. This finding might be related to the farmers’ unwillingness to reveal information about the present mortality and culling rate to researchers. The second-least understood statement was “complications from common procedures”. Complications could result in the reduction of feed intake or prolonged pain to the animal, which may precipitate poor welfare. Farmers showed little understanding of the statement and were probably unsure about its relationship to DCW. Moreover, pain detection in farm animals requires expertise and training, and such information may not have been available to the respondents. In contrast, farmers showed a better understanding of DCW when presented with a criterion such as physical appearance and cows’ response to handling procedures. These items are more situational and obvious manifestations of poor animal welfare [5]. For instance, physical injuries are easily detected during routine farm operations. Moreover, these items are in line with factors listed in Sabah’s Animal Welfare Enactment [14].

In this study, 9 (30.0%), 13 (43.7%), and 8 (26.7%) respondents were considered to have poor, satisfactory, or good knowledge about DCW, respectively, and showed that the knowledge differed among the farmers. A similar study conducted in the United States reported that dairy farmers showed significant variation in their understanding of cattle welfare [10]. Factors such as educational qualification, level of training, and farming system variables influenced the perception of DCW among cattle farmers in the United States [10], Brazil [9], and Bangladesh [19].

The factors associated with farmers’ knowledge about DCW criteria included educational qualification, animals’ production level, cattle breeds, and herd size. Nizam and Rahman [20] reported that a low level of literacy in Asia contributed significantly to the poor understanding of animal welfare. Clark et al. [13] also found that better-educated societies appeared to be more concerned and aware of farm animal welfare. The concept of animal welfare is commonly introduced in tertiary institutions or during specific training programs. Thus, farmers with better educational qualifications are more likely to have more access to information regarding animal welfare and understand the concepts. 

We also found that respondents with high producing cows and a large herd had higher knowledge scores compared to those with low-yield cows and small herds, respectively. This finding is in agreement with that of Kumar et al. [21] whereby farmers’ knowledge of DCW increased with their farm’s milk production. Accordingly, farmers or personnel involved in the management of large and intensive herds may be more exposed to welfare-related diseases or disorders such as mastitis and lameness. These conditions are commonly associated with large herds and high production levels [22,23]. In addition, farmers who were well trained and knowledgeable in DCW were able to detect clinical cases of mastitis and reduce on-farm prevalence [14]. Dairy cows use most of their energy for milk production, reproduction, and growth, which increases their susceptibility to diseases and disorders. An understanding of these events and challenges may prompt knowledgeable farmers to provide more care for their cows.

Farmers who kept crossbreeds had a higher knowledge about DCW compared to those that had only local breeds. This finding could be linked to the fact that most large dairy herds in Sabah have mainly exotic and crossbred cattle because they give more milk. Another reason could be the cost implications of poor animal welfare in exotic breeds because they are more likely to experience lameness, mastitis, or reproduction inefficiency compared to local breeds. Hence, the farmers who keep exotic breeds may prioritize DCW and identify the indicators of poor animal welfare better than those who have local breeds.

On the other hand, the frequency of veterinarian visits did not affect the farmers’ level of knowledge. This finding is in contrast to the reports by Wolf et al. [10] where local veterinarians had the second most influential role in influencing farmers’ knowledge of DCW. Other studies [24,25,26] reaffirmed that cooperation and communication between farmers and veterinarians could assist in improving animal welfare. This communication can take the form of education or herd-health programs, which allows veterinarians to be in contact with the farmers. Likewise, years of experience in farming was not associated with the farmers’ level of knowledge of animal welfare in this study. The lack of knowledge transfer between veterinarians and farmers regarding the topic may play a role in the finding.

The majority of the farmers also had a good opinion about DCW based on the criteria applied. They recognized that lower milk yield may be an indicator of poor DCW as they understood that reduction in milk production may indicate the presence of animal discomfort or stress. This assertion by the respondents is appropriate because common conditions such as mastitis and hoof lesions remain major components of poor welfare and may affect milk production. Furthermore, most of the respondents affirmed that the dry period is vital for ensuring optimal production in dairy cows. This was reflected as most of them disagreed with the statement, “cows should be milked during the last or two months of gestation”. The dry period allows the mammary gland to prepare for the next lactation and serves as an important period for the treatment and prevention of mastitis [27]. Furthermore, the vast majority of farmers, (93%, 28/30), understood that reduction in feed intake can be an indicator of poor animal welfare as it could ultimately lead to the reduction in a cow’s body condition score (BCS).

The important findings in this study regarding the farmers’ response to indicators of DCW were their position on the role of cows’ body condition, behaviour during milking, and hygiene. When presented with the DCW criteria, most of the respondents agreed that sudden changes in body condition indicated poor welfare. However, they did not recognize that cattle with a high body condition score may also show signs of poor farming practices. Dairy herds with a high body condition score can be predisposed to lameness [28], and reduced dry matter intake may further lead to periparturient metabolic disorders [29]. Furthermore, animals may respond to human handling in different ways, and it is considered to be an indicator of animal welfare. Dairy cows may respond positively to human handling, which is expressed as a reduction in flight distance [30]. On the other hand, a negative response is characterized by an increase in flight distance such as reluctance to enter a chute [30]. Farmers may not have an appropriate understanding of the topic as most failed to recognize that cows that express reluctance to enter the milking parlour or kicking during milking display potential signs of poor DCW. However, kicking, lifting and stepping during milking was associated with mastitis [27]. Specifically, stepping is related to the cow’s discomfort during milking and kicking indicates pain felt by the cow, probably from a teat lesion [27]. 

A high proportion of respondents (>70%) agreed that the hydration status of dairy cows was an indicator of DCW. Almost all of them (>90%) agreed that the presence of injuries was an indicator of poor DCW. In Brazil, deficiency in the provision of drinking water and the occurrence of hock injuries was found to affect local cattle [6]. Injuries to the limbs especially in the hock region were significantly correlated to the prevalence of lameness and could be an indicator of inadequate standards of comfort [8]. Likewise, most of the surveyed farmers agreed that the presence of excessive feces on the cows’ body indicated of poor DCW. The degree of manure contamination on the body, upper flank and limbs are used to assess hygiene [31]. Few studies reported that poor hygiene, characterized by excessive manure contamination of the body parts and limbs, was associated with a high prevalence of subclinical mastitis [32] and lameness [16], respectively. However, only 56% (13/30) agreed that reduced lying time is a sign of poor animal welfare. The lying down duration remains one of the most important ABMs in dairy cattle, and alterations in the behaviour have been associated with an increased risk of hoof injury [33] and reduced perfusion of the mammary gland [34].

Other items considered as indicators of DCW in this study included grooming and licking behaviour. Farmers seem to recognize them and the role they play in DCW because they classified behaviour into favourable and unfavourable groups. Excessive licking and grooming can be a sign of a mineral deficiency as cattle with a salt deficiency often show cravings or abnormal appetite for it [35]. They tend to lick various objects such as rock, wood, soil and even the sweat of other animals in the event of a salt deficiency. Calves without supplementary minerals would spend more time grooming, licking pen structures and ear sucking [36,37]. Napolitano [38] described the positive indications of mutual and self-grooming in dairy cattle and the negative indications associated with over-grooming, which includes high social tension. Mutual and self-grooming have been associated with hygiene, a comfortable living space and the reinforcement and stabilization of social relationships, which are integral aspects of behaviour in mammalian species [39,40].

Most of the farmers ranked their on-farm animal welfare status as good (57%), 37% considered theirs as satisfactory, while only 2 farmers described theirs as poor. This is in contrast with the results obtained regarding their knowledge about the criteria of DCW, where only 27% were considered to have a good understanding of the concept. Moreover, farmers had little understanding of the important indicators of DCW. Therefore, this reflects an underestimation of the animal welfare status on the respondents’ farm. To test such a hypothesis, future studies might consider comparing farmers’ and researchers’ welfare assessment of dairy cows.

This is the first study in the region to report the level of knowledge and attitude on dairy cattle welfare DCW among dairy farmers in Keningau, Sabah. In conclusion, the level of awareness about the topic needs to be improved. The majority of the farmers (70%) had satisfactory to good knowledge of DCW criteria; however, they need to be educated about the indicators of DCW, especially cows’ responses to human handling, behaviour during milking, and physical appearance. The factors observed that improved the farmers’ knowledge about DCW criteria included high education, large herds, high producing cows, and exotic breeds.

Limitations inherent in this study are well identified. The findings in this study are specific to dairy farmers located within the study region and the data are too small for any form of extrapolation to Malaysia dairy farms. The surveyed farmers were not randomly selected, as participation was based on their willingness and signing a consent form. Moreover, DCW is a broad aspect of the dairy industry, and the topics covered in the present survey require more elaboration for a better understanding of farmers’ opinions and knowledge about it. Nevertheless, this study serves as a preliminary work and an addition to our existing knowledge on the subject area in Malaysia dairies.

## 5. Conclusions

This is the first study in the region to report the level of knowledge and opinion on DCW among dairy farmers in Keningau, Sabah. In conclusion, the knowledge and opinion of DCW differed among the farmers in this study. This was evident in the disparity in understanding the basic criteria and indicators of DCW. Farmers with high education, large herd size, and high production levels showed a better understanding of DCW. Specifically, the farmers had a good understanding of it concerning changes in body weight, body condition and milk yield. However, their understanding and opinion on cattle behaviour and physical appearance needs to be improved.

## Figures and Tables

**Table 1:** Respondents’ and farm characteristics.

Variables	Respondents’ Characteristics	Frequency (*n*)	Percentage (%)
Gender	Male Female	22 8	73.3 26.7
Age	<30 year-old 30–49 year-old >50 year-old	13 11 6	43.3 36.7 20
Nationality	Malaysian Indonesian	25 5	83.3 16.7
Level of Education	Secondary Education Higher Education	23 7	76.7 23.3
Work Position	Managerial Non-managerial	15 15	50.0 50.0
Work Experience	<10 years >10 years	18 12	60.0 40.0
	**Farm characteristics**		
Management System	Semi-Intensive Intensive	3 27	10.0 90.0
Breed	Sabah Sahiwal Friesian (local breed)	21	70.0
	Crossbreed	9	30.0
Herd Size	Small (<50 cows) Medium (51–100 cows) Large (>100 cows)	11 13 6	36.7 43.3 20.0
Veterinarian visits	Rarely (once a year or less) Occasionally (more than once a year)	12 18	40.0 60.0
Animals’ Production Level (milk yield)	Low (<10 L/cow/day) Moderate (11–15 L/cow/day)	16 9	53.3 30.0
	High (>20 L/cow/day)	5	16.7

Note: Crossbred cattle were Jersey x Holstein–Friesian and Holstein–Friesian x Sahiwal.

**Table 2:** Mean knowledge score and number of respondents with poor, satisfactory, and good knowledge about DCW criteria.

Variables	Mean ± SD/Frequency	Percentage
Mean (±SD) knowledge score	12.1 ± 3.47	
Categories (cut-off values)		
Poor knowledge (<10.0)	9	30.0
Satisfactory knowledge (11–15)	13	43.7
Good knowledge (>15)	8	26.7

Note: SD = standard deviation

**Table 3:** Association between independent variables and the knowledge score of respondents regarding criteria of dairy cattle welfare.

		Knowledge Score		
Variables		Mean ± SD	*T*-Statistic/F Statistic	*p*-Value
Gender	Male	12.31 ± 3.59	1.21	0.08
	Female	14.25 ± 1.98		
Level of Education	Secondary	11.34 ± 3.52	−2.31	0.029
	Higher	14.57 ± 1.81		
Work Position	Managerial	13.13 ± 3.22	1.68	0.10
	Non-managerial	11.06 ± 3.49		
Breeds	Sabah Sahiwal Friesian	11.23 ± 3.23	−2.22	0.035
	Crossbreed	14.11 ± 3.29		
Age (years)	<30	11.53 ± 3.99	0.39	0.68
	30–49	12.81 ± 3.25		
	>50	12.00 ± 2.89		
Management system	Intensive	12.29 ± 3.58	0.36	0.93
	Semi-intensive	10.33 ± 1.52		
Herd Size (cows)	Small (<51)	11.58 ± 3.60 ^a^	4.05	0.02
	Medium (51–100)	11.00 ± 3.37 ^a^		
	Large (>100)	15.33 ± 1.21 ^b^		
Production level	Low	10.93 ± 3.37 ^a^	4.21	0.02
	Medium	12.22 ± 3.34 ^a^		
	High	15.60 ± 1.14 ^b^		
Frequency of Veterinarian visits	Once or less a year	12.36 ± 3.72	0.31	0.75
	More than once a year	11.94 ± 3.40		
Years of experience	<10 years	12.44 ± 3.88	0.52	0.66
	>10 years	11.58 ± 2.81		

Note: SD = standard deviation. Independent *t*-test and one-way ANOVA were applied for factors with 2 and 3 levels, respectively. Means with different superscripts are statistically different.

**Table 4:** Mean score of on-farm animal welfare status, far performance, and factors suggested to influence DCW.

Variables	Mean ± SD/Frequency	Percentage
Mean (SD) score selected by farmers	8.0 ± 1.83	
Farm performance (cut-off values)		
Good (<7.0)	17	56.7
Satisfactory (5–7)	11	36.7
Poor (>5)	2	6.7
	
Factors influencing on-farm DCW		
Farm facility	10	33.3
Farm workforce and human handling	5	16.7
Production	3	10.0
Management system		10.0
Health status	3	10.0
Miscellaneous	6	20.0

## Data Availability

The data presented in this study are available on request from the corresponding author.
